# *Orostachys japonicus* Inhibits Expression of the TLR4, NOD2, iNOS, and COX-2 Genes in LPS-Stimulated Human PMA-Differentiated THP-1 Cells by Inhibiting NF-*κ*B and MAPK Activation

**DOI:** 10.1155/2015/682019

**Published:** 2015-02-24

**Authors:** Yeo-Kwang Yoon, Hong-Jung Woo, Youngchul Kim

**Affiliations:** Department of Internal Medicine, College of Korean Medicine, Kyung Hee University, Seoul 130-872, Republic of Korea

## Abstract

*Orostachys japonicus* is traditionally used as an inflammatory agent. In this report, we investigated the effects of *O. japonicus* extract on the expression of genes encoding pathogen-recognition receptors (TLR2, TLR4, NOD1, and NOD2) and proinflammatory factors (iNOS, COX-2, and cytokines) in LPS-stimulated PMA-differentiated THP-1 cells and the NF-*κ*B and MAPK pathways. *O. japonicus* induced toxicity at high concentrations but had no effect at concentrations lower than 25 *μ*g/mL. *O. japonicus* inhibited LPS-induced TLR4 and NOD2 mRNA levels, suppressed LPS-induced iNOS and COX-2 transcription and translocation, and downregulated LPS-induced proinflammatory cytokine (IL-1*β*, IL-6, IL-8, and TNF-*α*) mRNA levels. In addition, *O. japonicus* inhibited LPS-induced NF-*κ*B activation and I*κ*B*α* degradation and suppressed LPS-induced JNK, p38 MAPK, and ERK phosphorylation. Overall, our results demonstrate that the anti-inflammatory effects of *O. japonicus* are mediated by suppression of NF-*κ*B and MAPK signaling, resulting in reduced TLR4, NOD2, iNOS, and COX-2 expression and inhibition of inflammatory cytokine expression.

## 1. Introduction

*Orostachys japonicus* (*O. japonicus*) is traditionally used as an inflammatory agent, antifebrile, homeostatic agent, and antidote and anticancer agent [1]. The methanol extract of* Orostachys japonicus *is thought to contain several different classes of phytochemicals, including triterpenes, sterols, and flavonoids [2]. Inflammation is caused by a variety of factors, including physical and chemical agents, the immune response, and tissue necrosis [3]. However, further studies on* O. japonicus* are required due to the lack of information on signaling pathways and physiological activity.

Immune cells can recognize pathogen-associated molecules, such as the lipopolysaccharide (LPS) of Gram-negative bacteria, the peptidoglycan (PGN) of Gram-positive bacteria, and mannans of yeast cells through toll-like receptors (TLRs) expressed on the cell surface [4]. Humans have various pathogen-recognition receptors including TLRs, nucleotide-binding oligomerization domain- (NOD-) like receptors (NLRs), and retinoic acid-inducible gene-1- (RIG-1-) like receptors [5–7]. These receptors transduce signals to activate nuclear factor-*κ*B (NF-*κ*B), which subsequently drives the induction of several proinflammatory cytokines and chemokines [8–10]. TLRs are an integral component of the inflammation process. TLR2 and TLR4, along with their ligands, are best characterized in terms of innate responses to bacteria, including* Chlamydia*. TLR2 is involved in the recognition of a broad range of microbial products, and TLR4 is the signal-transducing receptor for LPS [11]. NOD1 and NOD2 are involved primarily in mediating antibacterial defenses [12]. NOD1 recognizes mainly Gram-negative bacteria, whereas NOD2 recognizes most Gram-positive and Gram-negative bacteria [13].

Inducible nitric oxide synthase (iNOS) is expressed widely in various cell types and is highly expressed in LPS-activated macrophages [14]. Expression of the iNOS gene in macrophages is regulated mainly at the transcriptional level. NF-*κ*B is a pivotal regulator of important immunoregulatory genes involved in immune and inflammatory responses, including iNOS [15]. Cyclooxygenase-2 (COX-2) is expressed in the presence of many proinflammatory mediators, including LPS, interlukin-1*β* (IL-1*β*), and tumor necrosis factor-*α* (TNF-*α*), through which high concentrations of prostaglandin E_2_ (PGE_2_) are produced [16]. iNOS and COX-2 expression are regulated by NF-*κ*B. NF-*κ*B is a transcription factor that regulates several genes, including iNOS, COX-2, IL-1*β*, IL-6, and TNF-*α*, which are important for immunity and LPS-induced inflammation [17]. NF-*κ*B is activated by phosphorylation of inhibitory *κ*B*α* (I*κ*B*α*) through activation of mitogen-activated protein kinases (MAPKs), such as c-Jun N-terminal kinase (JNK), p38, and extracellular signal-regulated kinase (ERK)1/2 [18, 19]. In the present study, we investigated the effects of* O. japonicus* on the expression of genes encoding pathogen-recognition receptors (TLR2, TLR4, NOD1, and NOD2) and proinflammatory factors (iNOS, COX-2, and cytokines) in LPS-stimulated PMA-differentiated THP-1 cells, as well as the NF-*κ*B and MAPK pathways.

## 2. Materials and Methods

### 2.1. Extraction of* O. japonicus*

*O. japonicus* (20 g) was extracted by overnight incubation at 60°C in 500 mL of 80% methanol. The solution was filtered through Whatman No. 1 filter paper and concentrated using a rotary evaporator (Buchi, Flawil, Switzerland). The concentrated extract was freeze-dried (EYELA, Tokyo, Japan) and stored at 4°C in a vacuum container until use.

### 2.2. Cell Culture

Human monocytic leukemia THP-1 cells were supplied by the Korean Cell Line Bank. Cells were cultured in RPMI 1640 medium (GIBCO, Grand Island, NY, USA) containing 10% fetal bovine serum and antibiotics. Cells were incubated at 37°C in a humidified atmosphere of 5% CO_2_ in 95% air. THP-1 cells were treated with 100 nM of phorbol myristate acetate (PMA, Sigma-Aldrich Co., St. Louis, MO, USA) for 72 h to induce differentiation into macrophages. After differentiation, nonattached cells were removed by aspiration and adherent macrophages were washed with RPMI 1640 medium three times and then incubated in cell culture medium at 37°C.

### 2.3. Cell Viability

Cell proliferation was measured with CellTiter 96 Aqueous One Solution (Promega, Madison, WI, USA). Cells were seeded at 1 × 10^4^ per well in 96-well plates and incubated with different concentrations of* O. japonicus* at 37°C for 24 h, respectively. Cell viability was determined using a colorimetric assay with PMS/MTS solution. The absorbance was determined at 490 nm with background subtraction at 650 nm.

### 2.4. Treatment with* O. japonicus*

THP-1 cells were pretreated for 2 h in serum-free medium with* O. japonicus* (0–25 *μ*g/mL) and then incubated with LPS (1 *μ*g/mL) for 4 h (for mRNA expression) and 20 h (for protein expression). At each time point, total RNA and protein were isolated from the cultured THP-1 cells.

### 2.5. RNA Extraction and Real-Time PCR

Total RNA was purified from cultured cells using the TRIzol reagent following the manufacturer's protocol (Invitrogen, Carlsbad, CA, USA). First-strand cDNA synthesis was performed with 1 *μ*g of total RNA and it was transcribed to cDNA using a reverse transcription system with random hexamers (Promega) according to the manufacturer's protocol. The sequences for gene-specific primers were as follows: TLR2, 5′-TCTCCCATTTCCGTCTTTTT-3′ and 5′-GGTCTTGGTGTTCATTATCTTC-3′ (125 bp); TLR4, 5′-GAAGCTGGTGGCTGTGGA-3′ and 5′-TGATGTAGAACCCGCAAG-3′ (213 bp); NOD1, 5′-GTCACTGAGGTCCATCTGAAC-3′ and 5′-CATCCACTCCTGGAAGAACCT-3′ (363 bp); NOD2, 5′-CATGTGCTGCTACGTGTTCTC-3′ and 5′-CCTGCCACAATTGAAGAGGTG-3′ (226 bp); iNOS, 5′-TGGATGCAACCCCATTGTC-3′ and 5′-CCCGCTGCCCCAGTTT-3′ (59 bp); COX-2, 5′-CAAATCCTTGCTGTTCCCACCCAT-3′ and 5′-GTGCACTGTGTTTGGAGTGGGTTT-3′ (173 bp); IL-1*β*, 5′-TGATGGCTTATTACAGTGGCAATG-3′ and 5′-GTAGTGGTGGTCGGAGATTCG-3′ (140 bp); IL-6, 5′-GTGTTGCCTGCTGCCTTC-3′ and 5′-AGTGCCTCTTTGCTGCTTTC-3′ (194 bp); IL-8, 5′-GACATACTCCAAACCTTTCCAC-3′ and 5′-CTTCTCCACAACCCTCTGC-3′ (160 bp); TNF-*α*, 5′-ATCTTCTCGAACCCCGAGTG-3′ and 5′-GGGTTTGCTACAACATGGGC-3′ (51 bp); *β*-actin, 5′-GCGAGAAGATGACCCAGATC-3′ and 5′-GGATAGCACAGCCTGGATAG-3′ (77 bp). Real-time PCR was performed on the StepOneplus real-time PCR system with Power SYBR Green PCR Master Mix (Applied Biosystems, Foster, CA, USA). PCR was performed with 1 *μ*L of cDNA in 20 *μ*L reaction mixtures that consisted of 10 *μ*L Power SYBR Green PCR Master Mix, 2 *μ*L primers, and 7 *μ*L PCR-grade water. The reactions were performed with a denaturation step at 95°C for 10 min, followed by 40 cycles of 95°C for 15 sec and 60°C for 1 min. The crossing point of target genes with *β*-actin was calculated using the formula 2^−(target  gene−*β*-actin)^, and the relative amounts were quantified.

### 2.6. Western Blot Analysis

Cells were collected and washed with cold PBS and then lysed using lysis buffer (20 mM Tris-HCl (pH 7.5), 150 mM NaCl, 1 mM Na_2_EDTA, 1 mM EGTA, 1% Triton, 2.5 mM sodium pyrophosphate, 1 mM *β*-glycerophosphate, 1 mM Na_3_VO_4_, and 1 *μ*g/mL leupeptin) containing 1 mM PMSF (Cell Signaling Technology, Inc., Boston, MA, USA). The protein concentration was determined using a BCA protein assay according to the manufacturer's protocol. Protein (30 *μ*g) was fractionated by 12% SDS-PAGE and transferred by electrophoresis to nitrocellulose membranes. The membranes were blocked with 5% nonfat dry milk for 1 h at room temperature and then incubated overnight with antibodies against COX-2 (Santa Cruz Biotechnology, Santa Cruz, CA, USA), iNOS, NF-*κ*B p65, phospho-NF-*κ*B p65, I*κ*B*α*, phospho-I*κ*B*α*, JNK, phospho-JNK, p38 MAPK, phospho-p38 MAPK, ERK, phospho-ERK1/2 (Cell Signaling Technology), and *β*-actin (Sigma-Aldrich Co.), which were diluted to 1 : 1,000 with Tris-buffered saline containing 0.05% Tween 20 (TBS-T). After washing with TBS-T for 1 h, the membranes were incubated for 1 h at room temperature with horseradish peroxidase-conjugated secondary antibodies diluted to 1 : 2,500 in TBS-T. The membranes were subsequently washed with TBS-T for 1 h and proteins were detected using an Enhanced Chemiluminescence Kit (Thermo Scientific, Protein biology, IL, USA). Protein expression was analyzed using a Davinch-Chemi Chemiluminescence Imaging System (Davinch-K Co., Ltd., Seoul, Korea).

### 2.7. Statistical Analysis

Values were expressed as means ± SD. Student's *t*-test was used to evaluate differences between the LPS-only treated samples and LPS plus* O. japonicus* treated samples. ^*^ *P* < 0.05 and ^**^ *P* < 0.01 were considered to indicate statistical significance.

## 3. Results

### 3.1. *O. japonicus* Suppressed Cell Viability

**Figure 1:**
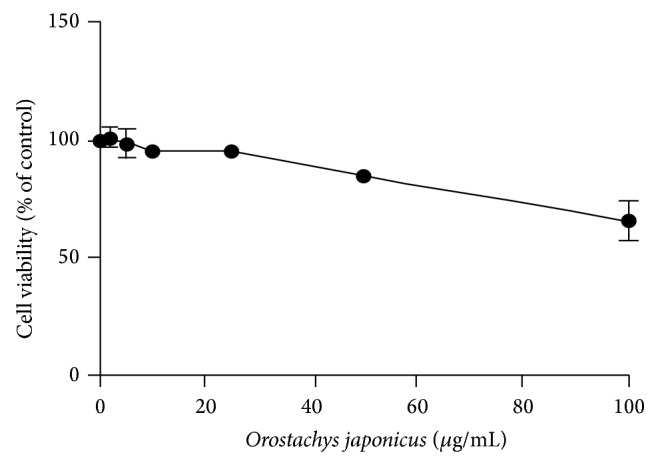
Effects of* O. japonicus* on the proliferation of THP-1 cells. Cells were treated with various concentrations of* O. japonicus* for 24 h. Cell viability was then determined by MTT assay. The data represent the means ± SD of triplicate samples.

The cytotoxicity of* O. japonicus* to THP-1 cells was examined by exposing cells to various concentrations of* O. japonicus* for 24 h. Cell viability was measured using the PMS/MTS assay.* O. japonicus* showed no toxicity at low concentrations (Figure 1).

### 3.2. *O. japonicus* Inhibited the LPS-Induced Expression of the TLR2, TLR4, NOD1, and NOD2 Genes

**Figure 2:**
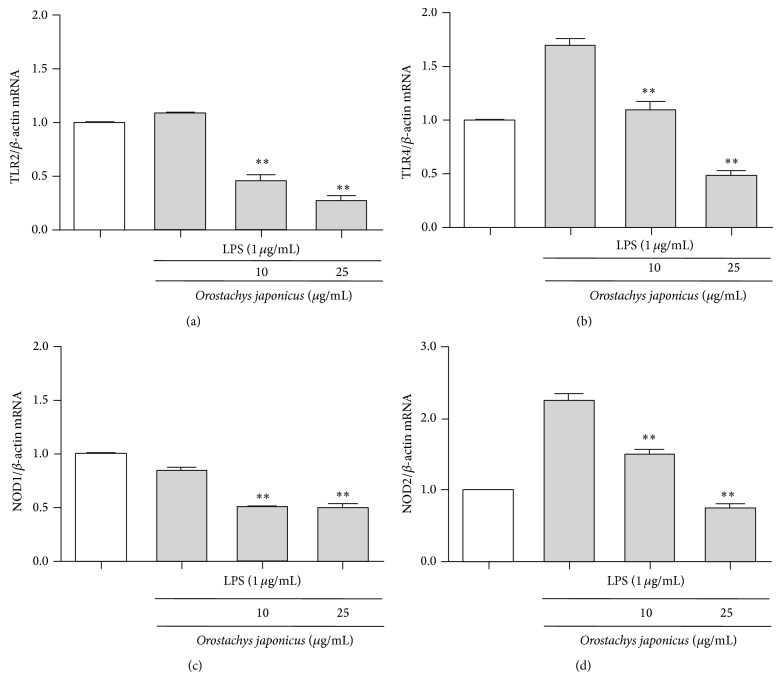
Effects of* O. japonicus* on LPS-induced TLR2, TLR4, NOD1, and NOD2 mRNA levels in THP-1 cells. Cells were pretreated for 20 h with various concentrations of* O. japonicus* (10 or 25 *μ*g/mL) before exposure to LPS (1 *μ*g/mL) for 4 h, and mRNA levels were measured using real-time PCR. The crossing points of the TLR2 (a), TLR4 (b), NOD1 (c), and NOD2 (d) with *β*-actin were entered into the formula 2^−(target  gene−*β*-actin)^, and relative amounts were quantified. The data represent the means ± SD of three independent samples. ^**^ *P* < 0.01 compared with LPS stimulation alone.

We investigated the effect of* O. japonicus* on the expression of TLR2, TLR4, NOD1, and NOD2 in THP-1 cells. mRNA levels were analyzed using real-time PCR. LPS induced TLR4 and NOD2 expression compared to the control.* O. japonicus* suppressed LPS-induced TLR4 and NOD2 transcription (Figures 2(b) and 2(d)). In contrast, TLR2 and NOD1 mRNA levels were not significantly affected after LPS stimulation (Figures 2(a) and 2(c)).

### 3.3. *O. japonicus* Inhibited LPS-Induced iNOS and COX-2 Gene Expression

**Figure 3:**
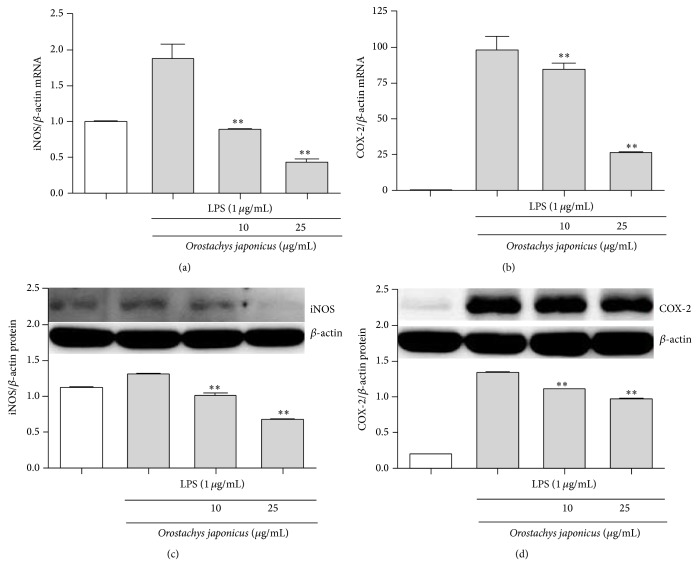
Effects of* O. japonicus* on LPS-induced iNOS and COX-2 mRNA and protein expression in THP-1 cells. Cells were pretreated for 20 h with various concentrations of* O. japonicus* (10 or 25 *μ*g/mL) before exposure to LPS (1 *μ*g/mL) for 4 h. Levels of (a) iNOS and (b) COX-2 mRNA were measured using real-time PCR. The crossing points of iNOS and COX-2 with *β*-actin were entered into the formula 2^−(target  gene−*β*-actin)^, and relative amounts were quantified. iNOS (c) and COX-2 (d) protein levels were determined by immunoblotting. Densitometric analyses are presented as the relative ratios of iNOS and COX-2 with *β*-actin. The data represent the means ± SD of three independent samples. ^**^ *P* < 0.01 compared with LPS stimulation alone.

We explored the effect of* O. japonicus* on inflammatory mediator expression. As shown in Figure 3, the response was dose-dependent, and the effect was significant at >10 *μ*g/mL* O. japonicus*. We evaluated whether* O. japonicus* affected iNOS and COX-2 mRNA transcription and protein levels. mRNA and protein levels were measured using real-time PCR and western blot analysis, respectively. LPS induced iNOS and COX-2 gene expression compared to the control.* O. japonicus* suppressed LPS-induced iNOS and COX-2 transcription (Figures 3(a) and 3(b)) and translation (Figures 3(c) and 3(d)).

### 3.4. *O. japonicus* Inhibited LPS-Induced IL-1*β*, IL-6, IL-8, and TNF-*α* Expression

**Figure 4:**
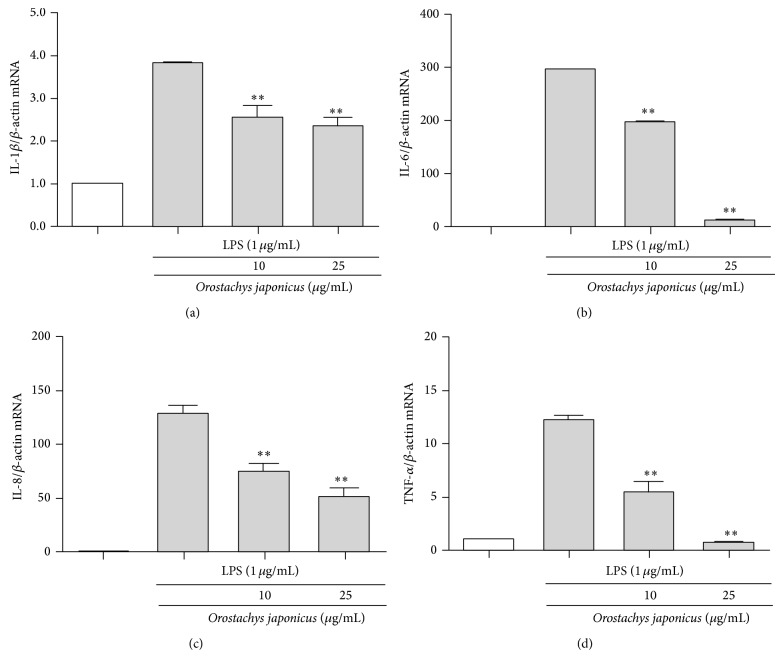
Effects of* O. japonicus* on LPS-induced cytokine mRNA levels in THP-1 cells. Cells were pretreated for 20 h with various concentrations of* O. japonicus* (10 or 25 *μ*g/mL) before exposure to LPS (1 *μ*g/mL) for 4 h, and mRNA levels were measured using real-time PCR. The crossing points of the IL-1*β* (a), IL-6 (b), IL-8 (c), and TNF-*α* (d) with *β*-actin were entered into the formula 2^−(target  gene−*β*-actin)^, and relative amounts were quantified. The data represent the means ± SD of three independent samples. ^**^ *P* < 0.01 compared with LPS stimulation alone.

We evaluated proinflammatory cytokine transcription in LPS-stimulated THP-1 cells. IL-1*β*, IL-6, IL-8, and TNF-*α* mRNA levels were evaluated using real-time PCR. LPS induced IL-1*β*, IL-6, IL-8, and TNF-*α* mRNA levels compared to the control.* O. japonicus* suppressed the LPS-induced IL-1*β* (Figure 4(a)), IL-6 (Figure 4(b)), IL-8 (Figure 4(c)), and TNF-*α* (Figure 4(d)) transcription.

### 3.5. *O. japonicus* Inhibited LPS-Induced NF-*κ*B Signaling and I*κ*B*α* Degradation

**Figure 5:**
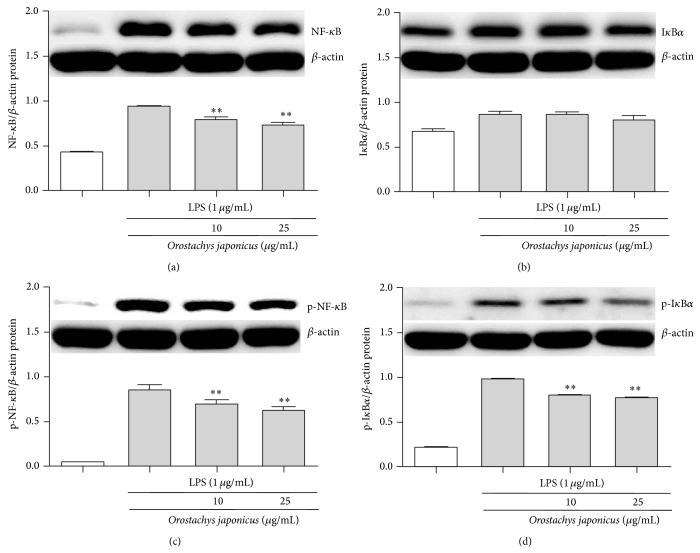
Effects of* O. japonicus* on LPS-induced NF-*κ*B p65 phosphorylation and I*κ*B*α* degradation in THP-1 cells. Cells were pretreated for 20 h with various concentrations of* O. japonicus* (10 or 25 *μ*g/mL) before exposure to LPS (1 *μ*g/mL) for 4 h, and NF-*κ*B p65 and I*κ*B*α* protein levels were determined using immunoblotting. Densitometric analyses were presented as the relative ratios of NF-*κ*B p65 (a) or p-NF-*κ*B p65 (c) and I*κ*B*α* (b) or p-I*κ*B*α* (d) to *β*-actin. The data represent the means ± SD of three independent samples. ^**^ *P* < 0.01 compared with LPS stimulation alone.

We next investigated the inhibition of NF-*κ*B p65 activity. The effects of* O. japonicus* on I*κ*B*α* degradation were examined. NF-*κ*B p65 and I*κ*B*α* protein levels were determined by western blot analysis. Treatment with LPS alone increased the phosphorylation of NF-*κ*B p65 and I*κ*B*α*, while* O. japonicus* inhibited LPS-induced phosphorylation of NF-*κ*B p65 (Figures 5(a) and 5(c)) and I*κ*B*α* (Figures 5(b) and 5(d)) in a dose-dependent manner.

### 3.6. *O. japonicus* Inhibited LPS-Induced Phosphorylation of JNK, p38 MAPK and ERK1/2

**Figure 6:**
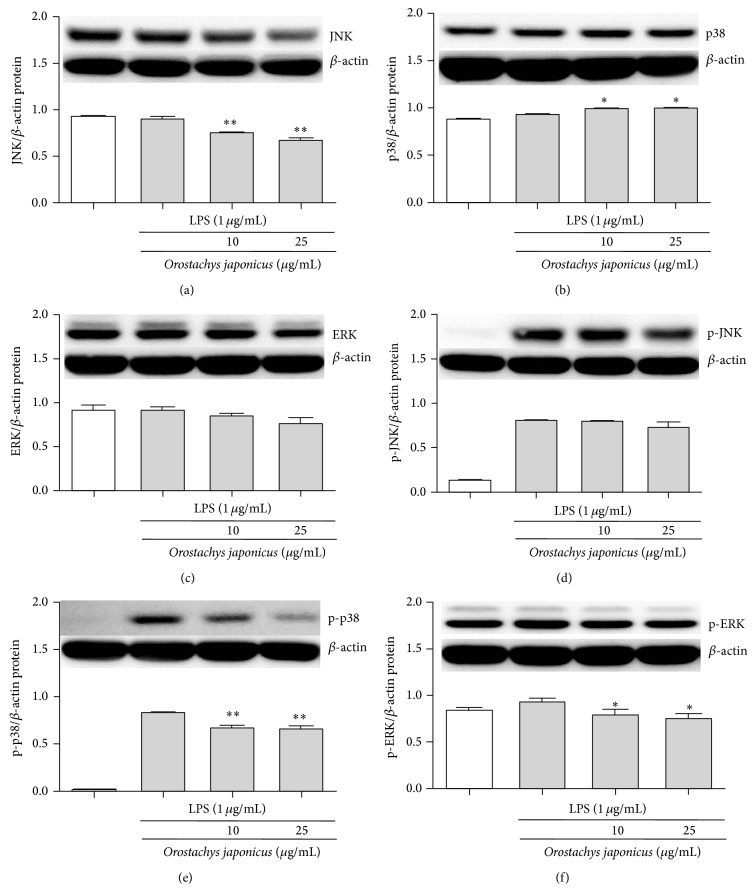
Effects of* O. japonicus* on the LPS-induced phosphorylation of JNK, p38 MAPK, and ERK in THP-1 cells. Cells were pretreated for 20 h with various concentrations of* O. japonicus* (10 or 25 *μ*g/mL) before exposure to LPS (1 *μ*g/mL) for 4 h, and JNK, p38 MAPK, and ERK protein levels were determined by immunoblotting. Densitometric analyses are presented as the relative ratios of JNK (a) or p-JNK (d), p38 MAPK (b) or p-p38 MAPK (e), and ERK (c) or p-ERK (f) to *β*-actin. The data represent the means ± SD of three independent samples. ^*^ *P* < 0.05 and ^**^ *P* < 0.01 compared with LPS stimulation alone.

To investigate the molecular mechanism underlying NF-*κ*B inhibition by* O. japonicus* in LPS-stimulated cells, we examined the effects of* O. japonicus *on the activation of JNK, p38 MAPK, and ERK1/2. MAPKs were assessed by western blot analysis. The phosphorylation of JNK, p38 MAPK, and ERK1/2 increased in cells treated with LPS alone.* O. japonicus* inhibited the phosphorylation of JNK (Figures 6(a) and 6(d)), p38 MAPK (Figures 6(b) and 6(e)), and ERK1/2 (Figures 6(c) and 6(f)) in LPS-stimulated cells in a dose-dependent manner.

## 4. Discussion

In the present study, we demonstrated that* O. japonicus *inhibits LPS-induced inflammatory signals in the PMA-differentiated human THP-1 cells. To evaluate the anti-inflammatory effects of* O. japonicus*, cells were pretreated with* O. japonicus* and then stimulated with LPS.* O. japonicus *exhibited dose-dependent cytotoxicity. Cell viability was not affected by 24 h treatment with less than 50 *μ*g/mL* O. japonicus*. Therefore, in the subsequent experiments 10–25 *μ*g/mL* O. japonicus *was used.

TLRs are expressed predominantly in monocytes/macrophages and neutrophils [20]. TLR2 and TLR4 are transmembrane receptors that transmit LPS signals to intracellular components in signal transduction pathways and play important roles in the immune system. TKR4 is associated with the recognition of Gram-negative bacterial LPS, and TLR2 is considered the receptor for Gram-positive bacteria [21]. While* O. japonicus *was shown to express both TLR2 and TLR4 in the present study, in the presence of LPS, TLR2 expression was higher than that of TLR4. Our findings suggest that* O. japonicus* could elicit inflammatory reactions in PMA-activated THP-1 cells and contribute to inflammatory processes, a process mediated by TLR4 and, to a lesser extent, TLR2. The peptidoglycan subunits are recognized by the NOD family proteins, in particular by NOD2. NOD2 is an intracellular protein involved in innate immunity and is associated with chronic inflammatory diseases in humans [22]. We found that* O. japonicus *downregulated LPS-induced NOD2 expression, with no effect on NOD1. Taken together, these results suggest that* O. japonicus *mediated inflammatory reactions through TLR4 and NOD2.

iNOS and COX-2 are important enzymes that mediate inflammatory processes and have been associated with the pathogenesis of certain types of human cancers, as well as inflammatory disorders [23]. Since proper regulation of iNOS and COX-2 expression could provide an effective and promising approach to treat inflammation related to diseases, much effort has been made to identify iNOS and COX-2 modulators, especially from plant sources [24, 25]. The present study demonstrates that* O. japonicus* extract can effectively suppress transcription and translational levels of iNOS and COX-2 expression. In addition,* O. japonicus* inhibited cytokine (IL-1, IL-6, IL-8, and TNF-*α*) expression in THP-1 cells stimulated by LPS. We evaluated transcription of proinflammatory cytokines, including IL-1*β*, IL-6, IL-8, and TNF-*α*, which play pivotal roles in the development and progression of inflammation, in LPS-stimulated cells. Our findings further suggest that* O. japonicus *possesses potent anti-inflammatory activity.* Harpagophytum procumbens *suppresses LPS-stimulated expression of iNOS and COX-2 in the fibroblast cell line L929 [26] and inhibits LPS-induced release of cytokines (IL-1*β*, IL-6, and TNF-*α*) and PGE_2_ from human monocytes [27].* Arisaema *cum bile extract inhibits the production of proinflammatory cytokines, including IL-1, IL-6, and TNF-*α*, and also inhibits iNOS and COX-2 expression, which are responsible for the production of NO and PGE_2_ in THP-1 cells [28]. The dichloromethane fraction from* O. japonicus* (OJD) inhibited NO production and TLR4, IL-1*β*, iNOS, and COX-2 expression in LPS-stimulated murine RAW 264.7 macrophage cells and inhibited LPS-induced NF-*κ*B p65 activation by suppressing I*κ*B*α* phosphorylation. However, phosphorylation of JNK and p38 MAPK was suppressed by OJD in a dose-dependent manner in LPS-stimulated cells [29]. Harpagoside inhibited LPS-stimulated NF-*κ*B promoter activity based on a gene reporter in RAW 264.7 cells, indicating that harpagoside interfered with the activation of gene transcription. These results suggest that the inhibition of iNOS and COX-2 expression by harpagoside suppresses NF-*κ*B activation [30]. Inhibition of the NF-*κ*B and MAPK pathways has been proposed to be a major mechanism underlying the attenuation of LPS-induced inflammatory cytokine production. NF-*κ*B plays a crucial role as the transcription factor in regulating many of the proinflammatory cytokine genes. LPS stimulation elicits a cascade leading to the activation of NF-*κ*B [31]. Cryptotanshinone suppressed LPS-induced production of IL-6 and TNF-*α* by inhibiting the activation of NF-*κ*B and MAPKs [32]. MAPKs, such as JNK, p38 MAPK, and ERK, mediate the signal transduction involved in cell proliferation, differentiation, transformation, survival, and death [33].

Expression of the NO, TNF-*α*, and IL-6 genes is dependent on activation of the transcription factor NF-*κ*B, which plays a crucial role in immune and inflammatory responses [34]. Activation of NF-*κ*B requires phosphorylation and proteolytic degradation of the inhibitory protein I*κ*B*α*, an endogenous inhibitor that binds to NF-*κ*B in the cytoplasm [35]. Upon stimulation with LPS, NF-*κ*B is activated and translocated into the nucleus as a result of phosphorylation-mediated degradation of I*κ*B*α* proteins in the lung of AL1 mice. However, pretreatment with a suitable drug could decrease the degradation of I*κ*B*α* and nuclear translocation of NF-*κ*B p65 and, therefore, downstream TNF-*α* and IL-6 production. However, MAPKs including JNK, p38 MAPK, and ERK play an important role in signal transduction pathways and regulate cytokine release [36]. In this study, MAPK was activated in LPS-induced THP-1 cells. However, drug treatment markedly suppressed LPS-induced phosphorylation of JNK, p38 MAPK, and ERK. The inhibition of IL-1*β*, IL-6, IL-8, and TNF-*α* production by* O. japonicus *occurs through pathways that converge on p38 MAPK and I*κ*B*α* activation since these kinases are known to regulate cytokine production in LPS-induced THP-1 cells.* O. japonicus *inhibits anti-inflammatory responses by inhibiting the degradation of I*κ*B*α* and nuclear translocation of NF-*κ*B and downstream cytokine expression. These results suggest that drug activity was dependent in part on the inhibition of MAPK and NF-*κ*B signaling pathways.

## 5. Conclusion

In this study, we found that treatment with* O. japonicus* blocked the activation of JNK, p38 MAPK, and ERK1/2, suggesting that* O. japonicus* suppresses LPS-induced NF-*κ*B translocation by inhibiting the activation of these intracellular signaling cascades and reducing iNOS and COX-2 expression.
